# Immediate Needs and Concerns among Pregnant Women During and after Typhoon Haiyan (Yolanda)

**DOI:** 10.1371/currents.dis.29e4c0c810db47d7fd8d0d1fb782892c

**Published:** 2016-01-25

**Authors:** Mari Sato, Yasuka Nakamura, Fumi Atogami, Ribeka Horiguchi, Raita Tamaki, Toyoko Yoshizawa, Hitoshi Oshitani

**Affiliations:** Department of Maternal Nursing, Tohoku University Graduate School of Medicine, Sendai, Miyagi, Japan; Women’s Health Nursing, Tohoku University Graduate School of Medicine, Sendai, Miyagi, Japan; Women’s Health Nursing, Tohoku University Graduate School of Medicine, Sendai, Miyagi, Japan; Maternal and Child Center in Takaishi City, Takaishi, Osaka, JapanTakaishi-shiritsu maternal and child health birthing center; Department of Virology, Tohoku University Graduate School of Medicine, Sendai, Tohoku, Japan; Women’s Health Nursing, Tohoku University Graduate School of Medicine, Sendai, Miyagi, Japan; Department of Virology, Tohoku University Graduate School of Medicine, Sendai, Miyagi, Japan

## Abstract

Introduction: Pregnant and postpartum women are especially vulnerable to natural disasters. These women suffer from increased risk of physical and mental issues including pregnant related problems. Typhoon Haiyan (Yolanda), which hit the Philippines affected a large number of people and caused devastating damages. During and after the typhoon, pregnant women were forced to live in particularly difficult circumstances. The purpose of this study was to determine concerns and problems regarding public health needs and coping mechanisms among pregnant women during and shortly after the typhoon.

Methods: This study employed a cross-sectional design utilizing focus group discussions (FGDs). Participants were 53 women (mean age: 26.6 years old; 42 had children) from four affected communities who were pregnant at the time of the typhoon. FGDs were conducted 4 months after the typhoon, from March 19 to 28, 2014, using semi-structured interviews. Data were analyzed using the qualitative content analysis.

Result: Three themes were identified regarding problems and concerns during and after the typhoon: 1) having no ideas what is going to happen during the evacuation, 2) lacking essentials to survive, and 3) being unsure of how to deal with health concerns. Two themes were identified as means of solving issues: 1) finding food for survival and 2) avoiding diseases to save my family. As the pregnant women already had several typhoon experiences without any major problems, they underestimated the catastrophic nature of this typhoon. During the typhoon, the women could not ensure their safety and did not have a strong sense of crisis management. They suffered from hunger, food shortage, and poor sanitation. Moreover, though the women had fear and anxiety regarding their pregnancy, they had no way to resolve these concerns. Pregnant women and their families also suffered from common health problems for which they would usually seek medical services. Under such conditions, the pregnant woman cooperated with others for survival and used their knowledge of disease prevention.

Discussion: Pregnant women experienced difficulties with evacuation, a lack of minimum survival needs, and attending to their own health issues. Pregnant women were also concerned about needs and health issues of their families, particular, when they had small children. Collecting accurate information regarding the disaster and conducting self-sustainable preparation prior to the disaster among pregnant women will help them to protect their pregnancy status, thereby improving their families’ chance of survival during and after disasters.

## Introduction

Pregnant and postpartum women with infants are especially vulnerable to natural disasters.^1^^,^ 2 ^,^ 3 These women suffer from increased risk of lower genital tract infection and inflammatory diseases following earthquakes,^4^ and disasters are associated with premature rupture of membrane, premature labor, and delivering low birth weight babies.^5^^,^ 6 Moreover, women’s status in society may render them more vulnerable to disaster-related economic impacts, which may result in decreased access to healthcare resources.^1^

Pregnant women are also vulnerable to mental health issues due to the considerable stress experienced during a disaster.^7^Existing anxiety about earthquakes and parity were found to be significantly correlated with postnatal depression,^8^ and perceived stresses such as pressure from parents’ roles, concerns about maternal and child health and their body, and physical activity changes resulting from pregnancy are significantly correlated with both depression and post-traumatic stress disorder.^9^ Thus, pregnant women are more likely to suffer both physical and mental health problems as compared to the general population during and after disasters.

Asian countries are particularly vulnerable to natural disasters. From January 2001 to December 2010, there were 38,400 disasters registered in the disaster database.^10^ Of the deaths from these disasters, 62.5% occurred in Asia.^11^ This is because major disasters commonly occur in Asia, and during this period, some major disasters occurred, such as Indian Ocean tsunami, the cyclone in Myanmar, and the Sichuan earthquake. The Philippines is one of the most disaster-prone countries among Asian countries,^12^ and it is identified as one of 10 nation-states most vulnerable to climate change.^13^ Typhoon Haiyan (local name “Yolanda”), which struck the central part of the Philippines on November 8, 2013, was one of the strongest typhoons in history, affecting 25 million people.^14^ The damages were particularly severe in the Eastern Visayas Region, which includes three main islands: Leyte, Samaar, and Biliran. A total of 6,201 deaths and 28,626 injured people have been confirmed, and 1,785 people are still missing.^14^ Many others have lost their homes. Most human casualties were a result of the storm surge, and the number of casualties was particularly high in municipalities along the coast of Leyte Gulf.^15^ After Typhoon Haiyan, the World Health Organization (WHO) identified the problems associated with pregnancy and childbirth as important health issues.^16^

A study exploring pregnant women’s experiences during Hurricane Katrina showed that women were comforted by the knowledge of necessary information to survive, and they indicated that social support, jobs, homes, and the comfort of their life plan were the most significant losses, which led to uncertainty and feelings of depression.^17^ Moreover, pregnant women and infants have increased health concerns due to disruption of basic sanitation, as well as inadequate access to clothing, food, and housing.^18^ Thus, pregnant women are greatly affected by disasters, as they are not only burdened by their own physical and mental problems, but also their children and family’s livelihood problems to survive and protect their health. In particular, the Philippines is still ranked around the middle in mothers’ index rankings, and maternal and under-5 mortality rates remain high.^19^ Accordingly, pregnant women in the Philippines might be forced to live in particularly difficult circumstances during a disaster. Therefore, it is crucial to learn lessons from pregnant women’s experiences under extraordinary circumstances during and after the disaster.

The purpose of this qualitative study was to determine the concerns and problems regarding public health needs of pregnant women during and soon after Typhoon Haiyan. Moreover, we documented how pregnant women coped with their problems. We also identified common problems that occurred among pregnant women to improve preparedness and resilience at the community and local government levels, including preparedness of local health facilities. These findings may also include some suggestions for partners that provide external supports after disaster with regard to what they should prepare and how they should operate with an understanding of pregnant women’s needs during and after mass-casualty events.

## Methods

**Design**

This study was conducted as a cross-sectional study using focus group discussions (FGDs).

**Study Area**

We selected four communities from three municipalities—two communities from the inland and two communities from coastal areas of Leyte Province, which was the most severely affected province. Although most of the deaths and injuries occurred in coastal areas, most houses in both coastal and inland areas were either partially or totally damaged. This discrepancy between human casualties and household damages can be explained by the fact that most human casualties were caused by the storm surge, while household damages were caused by both storm surge and extremely strong winds. The study sites were determined by suggestions from the Leyte provincial health office, and they include both coastal and inland communities considering the above-stated discrepancy in typhoon damage.

Background information of the three municipalities is shown in Table 1. We selected two communities from Municipality X; one from the coastal area and another from the inland area. Municipality Y was located in the inland area and Municipality Z was located along the coast. The number of deaths and missing persons was significantly higher in Municipality X, resulting from it being hit directly by the storm surge, though the household damages showed no differences among the three municipalities. The disaster impacts on the health facilities, including the Rural Health Unit (RHU), were almost the same; classified as “damaged the roofs.”

**Participants**

Participants included 53 women who were pregnant at the time of the typhoon. Participants were recruited via purposive sampling, with the help of the public health nurses (PHNs) and midwives working at the rural health units in Leyte Province. The enrollment criteria of the participants included the following: 1) women aged over 20 years old, 2) women who were pregnant regardless of gestation period when Typhoon Haiyan occurred, 3) women who were capable of talking about their experiences during and after the typhoon without emotional turmoil, and 4) women who consented to the FGDs.

Pregnant women were invited to participate in the study after they were given an explanation of the study’s objectives by the PHNs and midwives.

**Data collection**

FGDs took place 4 months after the typhoon, from March 19 to 28, 2014, using semi-structured interviews. Each discussion lasted about 60–70 minutes. The time and place of the FGDs were arranged according to availability of the local government offices. Initially, broad questions about the situation of women and their families when the typhoon hit and immediate public health problems and concerns after the typhoon (on the day of typhoon, during 1 week, and during 1 month) were asked (Appendix 1). We then focused on how they acted to address their problems. When necessary, the interviewers prompted the interviewees for further explanation. All FGDs were conducted in Waray-Waray (the local language of Leyte Province).

The interviewers included three female registered nurses aged 28 and 29 years old who were able to speak three languages: English, Tagalog, and Waray-Waray. Before starting the FGDs, the research group members explained the research objectives to them and trained them on methods of how to organize FGDs. They also underwent a 1-day simulation-based training for conducting FGDs. All FGDs were conducted by all three interviewers with different assigned responsibilities: the main interviewer, assistant interviewer, and recorder. Two or three research group members always supervised the FGDs, and one was fluent in Waray-Waray. After each FDG, research group members and interviewers discussed what they found to generate a common understanding on major findings of FGDs. There were four FGD sessions, and the number of participants for each session was 15, 8, 17, and 13 women, respectively.

**Data analysis**

We used the qualitative content analysis approach, which is widely used in qualitative health studies. The goal of this analysis was to identify important themes or categories within a transcript. It allowed the research group members to understand context in a subjective but still scientific manner.^20^ Among three common approaches for qualitative context analysis—conventional, directed, and summative—we chose the conventional analysis approach, which aims to describe a phenomenon. By using this approach, it is possible to obtain the categories and names for categories from the data without preconceived categories, which is also described as inductive category development.^21^ As this study extracted the concerns and problems from the phenomenon during and after the disaster using raw data of the FDGs, we believed that this approach was appropriate to reveal the categories of real problems faced by our participants and their concerns.

One interviewer listened to the recordings, which were in the Waray-Waray language, and transcribed them verbatim. Subsequently, the interviewer translated the transcripts into English. The accuracy of the translation was checked by another interviewer, who back-translated the English into Waray-Waray. Subsequently, all interviewers re-confirmed the data by listening to the recordings once again before finalizing them. The research group members also read the English transcripts and resolved any ambiguities by consulting the interviewers.fter they identified the codes and sub-categories, the other two research group members joined in the discussion to finalize them. To ensure overall credibility, conformability was assessed by examining the degree of agreement among the research group members.

First, two research group members read each FDG transcript in-depth several times and extracted codes and subcategories by an inductive process: important sentences and phrases were picked up, and main ideas derived from them were identified as codes. Then, the analysis unit was established in which the transcripts were read again to ensure that no codes were left out from the previous process and to re-examine the subcategories. Subsequently, similar sub-categories were identified and used to construct themes. Some of the themes were combined to extract the main concepts. Finally, three themes were identified regarding the problems and concerns during and after the typhoon, and two themes were identified as means of solving these problems and concerns.

To minimize bias, the two research group members met periodically to discuss the analytic process and findings. After they identified the codes and sub-categories, the other two research group members joined in the discussion to finalize them. To ensure overall credibility, conformability was assessed by examining the degree of agreement among the research group members.

**Ethical considerations**

Ethical approval was obtained from the Ethics Committee of Tohoku University Graduate School of Medicine (No. 2013-1-251). Participants were asked to sign a consent form prior to participation in the FGDs, and were informed of the risks and benefits associated with participation. As a follow-up measure, the authors visited the commune chiefs 1 week after the FGDs to check on the participants’ emotional wellbeing as a result of remembering the disaster after the FGDs.

## Results

Among 53 participants, most them were in their 20s (mean age: 26.6, range: 16-44). Eleven (20.8%) were primigravida and the rest (79.2%) were multigravida. Nine (17.0%) had one child, 13 (24.5%) had two children, eight (15.1%) had three, and 12 (22.6%) had four or more before the pregnancy during the typhoon. About their pregnancy, 24 (45.3%) were in the first trimester, 14 (26.4%) were in the second trimester, and 15 (28.3%) were in the third trimester. All participants suffered extensive material losses from the typhoon. The houses of 47 participants (88.7%) were totally destroyed by the storm surge and/or strong wind of the typhoon.

Three themes were identified for problems and concerns during and after the typhoon: 1) having no idea what is going to happen during the evacuation, 2) lacking essentials to survive, and 3) being unsure of how to deal with health concerns. Regarding means of solving issues, two themes were identified: 1) finding food for survival and 2) avoiding diseases to save the family.

**1. Problems and concerns**

**1-1. Having no ideas what is going to happen during the evacuation**

***I made a mistake in judgment regarding the need to evacuate***

Although most pregnant women recognized that the typhoon was coming, some did not recognize the need to evacuate. The pregnant women already had several typhoon experiences, and they had never had any major problems previously. For that reason, they underestimated the catastrophic nature of this typhoon. “Health volunteers disseminated the information for evacuation. But we did not care about it, and we just stayed inside our house.” “Our house already experienced many typhoons, but it remained standing. For this reason, we were just comfortable resting inside.” However, after the wind and the rain became intense, the women noticed something extraordinary and made a quick judgment that they should escape. “It was 8 o’clock in the morning when the rain started heavy. I thought that my house would be damaged, and I decided to evacuate with my children.”

***Evacuation shelters were exposed to danger***

Some pregnant women decided to evacuate before the typhoon came, and they chose buildings with more resistant materials compared with their houses, such as concrete buildings, as they believed these places were secure. “During the storm, we escaped to the police officer’s house because the house was made of concrete materials.” Unfortunately, the typhoon’s intensity was beyond expectations, and the women again had to escape to search for the safest possible place with their families again. The women needed to seek an escape route while trembling with fear. There were no safe places until the typhoon had passed. “Glass windows were broken like after bomb explosion in the house we evacuated. We moved to another house after the roof in the kitchen fell down. We ran again to the other side.” “We were in the evacuation shelter. The wind grew strong, and we were afraid because the roof materials flew overhead. We ran into the toilet room.”

***I first experienced feeling that death was certain***

The pregnant women were in great peril when the typhoon hit their locations. The typhoon destroyed many buildings, and the women who evacuated and those who did not had an encounter with imminent danger that they had not previously experienced. “I started to cry hard because I thought that this might be the end of my life.” “We did not expect that we could survive because only the terrace was left.” Some of the pregnant women were injured during the evacuation, and they ran around with gravid bodies. “While I was crossing the school ground, the strong wind occurred again and it was like I was being pushed by the air. I fell down again and I hit my back with a hard object.”

***I was confused by mixed rumors***

The pregnant women were bombarded with contradictory information. Increasing fear shut off the women’s rational minds and removed the clear distinction between right and wrong. Some believed that a tsunami might be coming, and they had to run from a renewed sense of fear. “Someone who rode a motor bike and roamed around the community informed us that a tsunami would be coming. We ran to the mountain and some went to the church.” “Someone told us that a tsunami would hit us. We ran to the third floor.”

***I suffered from possibility of preterm labor***

Some pregnant women forgot about their pregnancy, and the others experienced abnormal pregnancy symptoms during evacuation. Though they worried about these abnormal signs, they had no choice but to endure them. “During the typhoon, I felt some contraction and bloody spots.” “I did not really care about the pregnancy during the typhoon. I kept on running.” Some of them prepared themselves for losing their babies. “I could not bear the pain anymore. I thought that I would terminate this pregnancy.”

**1-2. Lacking essentials to survive**

***Nothing I could do as a mother when my children suffered from hunger and limited food***

Soon after the typhoon passed, children started complaining of hunger. However, the pregnant women could not provide any food for their children, and they felt terrible seeing their children starving. “Soon after my children evacuated to the church, they were already starving.” “My kids were hungry around 7 o’clock in the morning.”

***We had no choice but to eat wet, smelly rice***

The pregnant women looked for any food they could eat and give to their families. Some found rice, but it was wet and smelly because it was soaked in dirty water, and they were hesitant to eat it. However, they needed any available food, and thus they had only one option. “To endure our difficult situation, we cooked even foul-smelling rice. We had no other choices.” Further, some pregnant women who had babies could not obtain baby formula, leading to further difficulties. “Even my 1-year-old baby drank Royal (soft drink). We were not able to loot some milk for my baby.” “Babies just ate the coconut meat, like adults, because we had no choice.”

***We just stayed in freezing cold, after losing all belongings***

Because of the storm surge and rain, the pregnant women and their families were completely drenched. The roofs were blown off by a strong wind, and they lost all of their belongings, most notably dry clothes. “I felt so cold, and I looked for dry clothes.” “After the typhoon, we had nothing. All things were wet and damaged.”

***Concerns with defecation because of toilet dysfunction***

Most of the toilets lost their roofs and water for flushing. In the evacuation center, the pregnant women shared one toilet without any water. They just had to use a dirty toilet that was covered by urine and stool. “Some people defecated on the floor in the evacuation center. I told my husband to flush the toilet, as there was a lot of poop all over the floor. I had no choice. I just covered my nose while using the toilet.”

**1-3. Being unsure of how to deal with health concerns**

***We had skin problems, diarrhea, and leptospirosis caused by the storm surge and unsanitary conditions***

The pregnant women complained of health concerns regarding skin diseases, and they thought this was because of the effects of the storm surge. “It was like a burn that caused some blisters. When the skin peeled off, it bled.” “We had ‘ata,’ which is a waterborne disease on the feet that came from the storm surge.” They also suffered from diarrhea, even though the women were sensitive to avoid diarrhea by using boiled water. “We made sure to boil the water properly for children to avoid the occurrence of illness.” Some family members got leptospirosis, and this was worsened in causing weakness and an inability to stand up. “My brother got leptospirosis by rat urine. He felt weak and could not even stand. We sent him to the doctor.” The pregnant women believed that all these symptoms occurred because of unsanitary conditions after the storm surge.

***I developed physical concerns that required medical services***

The pregnant women suffered from common health problems for which they would usually seek medical services. “My mother and father got a fever.” “My child had asthma.” “The health problems among my family were cough and cold.” They had to find a way out for them, as the health facilities were severely damaged and the health system was disrupted. “We sent our children to another province to get medication. Children could only take oral rehydration solution because there were medical services in their community.”

***I was uncertain of my pregnancy status due to unavailability of medical care***

Although the pregnant women had a number of symptoms, they could not receive antenatal care until 1 month after the typhoon. “I could not move properly because I was suffering from some pain on my stomach.” “I felt some pain in my tummy during the typhoon.” The women had to act by making their own decisions to either walk a long way to the health centers or wait for medical services to come to their communities. “I had my prenatal care 1 month after the typhoon, in December. I just went directly to the health center, and other mothers did not come with me.”

The pregnant women had to make decisions for seeking health services by themselves. Most health facilities had been destroyed, and external medical assistant teams supported the facilities first, and thus they did not reach out to the communities for several months. “Nobody came for the pregnant women. We went directly to the rural health unit.”

***We suffered from acute stress disorder***

The pregnant women and their children became nervous with heavy rain and wind after the terrifying experiences. They certainly could not forget that nightmare-like day. “Most of children here had trauma. When they saw dark clouds, they feared that the Typhoon Haiyan would be coming to hit us again.” “Until now, I still feel nervous, when it rained hard just like yesterday.”

**2. Means of solving issues**

**2-1. Eat for survival**

One of the primary needs for the women was to get something to eat for their families. They asked their husbands and children to find any food possible, and family members moved around the communities where everything had been lost and buildings had collapsed. “Our husbands walked as a group to find food, even in small amounts.” Some of them found some shops to buy food. “We went to a grocery store to get some food, but that lasted for 3 days.”

The pregnant women also distributed and shared any food, even in small amounts, among the evacuees. The damage of the typhoon made daily life more difficult for everyone. They recognized that helping each other was most beneficial for survival as an unconscious instinct. “If someone had water, they shared with us.”

Some women’s families resorted to any means necessary to obtain food for their children. They stole goods to save their lives under the pressure of the crisis. “My husband looted some formula for my baby. By grace of him, I could bottle-feed my baby with that milk.” “Some others were looting rice.”

As opposed to the food situation, water was not as scarce. The pregnant women found water from wells, which could be found on the mountainside and in rural areas. Fortunately, some wells were not directly affected by the storm surge and remained fully functional. “We lived on the mountainside, and the storm surge did not reach that area. Our deep well was functional.”

**2-2. Avoiding diseases to save the family**

The pregnant women realized that it was necessary to boil the water before giving it to their children. This might have helped to avoid some illnesses. They always boiled drinking water, as this was the only way they could prevent disease without any assurance of medical services. “We made sure to boil water properly for children to avoid the occurrence of illness because there was no medicine available.”

To resolve toilet problems, the pregnant women shared the toilets with neighbors or relatives. Because of the wind, most of the toilets had lost their roofs, despite retaining their function. “The toilet was affected by fallen debris. I cleaned it, and I tried to flush. Luckily, it was still working, and all families used this toilet.” Some participants used the river as a toilet. “We defecated in the river. At least the water was free flowing in the river.”

The pregnant women tried to obtain health services somehow on their own. Some of them sent their family members to retrieve medication from other communities, and others solved their health concerns just by taking drugs. “My husband was able to loot some medicine, which we took.” Some pregnant women received information on the restart of the immunization program at the RHU from the RHU health staff and the health services from nongovernmental organizations. “A nurse came here to inform us that RHU had a free anti-TT injection.”

## Discussion

During Typhoon Haiyan, many of the pregnant women could not make the correct decision to evacuate. They initially thought that it was not necessary to evacuate based on their past experiences with typhoons, and then, they became confused by a completely unfounded rumor—that there was an impending tsunami. Although they managed to evacuate before the storm surge, they had difficulty finding a safe place for shelter. This suggests that the pregnant women underestimated the risk of a disaster and did not have a strong sense of crisis management. Warsini et al.^22^ suggested that people in areas seriously damaged by a volcanic eruption tended to ignore the necessity for evacuation or to delay evacuation decisions. Moreover, those who had never experienced multiple losses before were less likely to have coping and emotional skills necessary to deal with a disaster. Thus, evacuation preparedness for disasters should include a variety of realistic scenarios and plans for the worst-case scenario. This would increase awareness of the need to evacuate even for people who do not have prior experiences. T he disaster preparedness planning and rapid response capacity among community members can minimize disaster damages.^23^ It is important to develop strategies for involving community members in taking action in future crises.^24^ It is also critical to obtain accurate information to make the right decision to evacuate. Disaster mitigation strategies of the community should include a mechanism to obtain and disseminate accurate information to all residents.

Pregnant women faced a serious health risk associated with their pregnancy and experienced anxieties about their pregnancy. Although pregnancy is different from diseases, pregnant women are at higher risk of adverse events than healthy adults are. Pregnant women are exposed to increased risk of miscarriage, premature delivery, and giving birth to low birth weight babies during and after disasters^5^, and our participants noticed pregnancy-related symptoms such as abdominal pain and genital bleeding as well as an inability to handle them. To minimize the risk for pregnant women during a disaster, educational programs should be provided to pregnant women regularly as a component of disaster preparedness. A study evaluating the effectiveness of educational programs for disaster preparedness for pregnant women found several effective interventions, including establishing a contact method with families during disasters, receiving medical examinations such as antenatal care during disasters, and preparing for emergency.^25^ Unfortunately, we did not find any benefits from existing training programs that were offered to pregnant women before the typhoon. Training on how to deal with pregnancy-related symptoms during a crisis should be provided during prenatal care. This would allow pregnant women to take responsibility for their pregnancy and develop coping strategies for disasters.

Adequate social support may also help pregnant women cope with a stressful situation and/or minimize the stress reaction. A study on the effects of displacement due to flooding during pregnancy on birth outcomes showed that perceived social support had a positive effect on infant birth weight among displaced women.^26^ Women with multiple types of support from different sources, including family, husband, and society, during pregnancy give birth to infants with higher birth weights.^27^ In this study, we found that the pregnant women shared meals among evacuees and neighbors. Acute shortages of food and drinking water caused severe uncertainty for the pregnant women, and alleviating this worry might reduce anxiety to allow for better maintenance of physical and mental health. Thus, pregnant women should communicate well with people around them during normal times and inform them of their needs to obtain adequate support.

Pregnant women also suffered from life-threatening risks due to a lack of essential needs for survival, such as acute shortages of food, safe drinking water, and clothes immediately after the typhoon. They shared available food and searched for food by all means possible in order to survive. Fortunately, water was readily available because wells remained operational. However, they did not know when the relief goods, including food, would arrive. The humanitarian emergency response must improve,^28^ and emergency response agencies should consider the themes elicited from the community residents when providing immediate support and long-term relief.^29^

Pregnant women were concerned about needs of their families as well as their own, particularly when they had small children. Women with small children usually need essential support and those supports are important to maintain their daily living functions^30^. They commonly receive supports from their partners and other relatives, which may not be available during and after a disaster. Household tasks including taking care of their family and children also continue to be their tasks during and after a disaster. Pregnant women who already had small children in this study had difficulty finding formula milk and providing a proper weaning diet. Health personnel should collaborate with community residents and other professional groups to educate mothers and their families about concrete disaster preparedness regularly. Such education should include the need for stockpiling less commonly available nutrition such as formula milk and weaning food.^31^ Breastfeeding has been demonstrated as the most appropriate method for delivering nutrition in a disaster situation, as it is safe and can be given at any time without need for hot water^32^ and under suboptimal hygiene conditions.^18^ To that end, pregnant women must recognize the importance of giving breast milk and of consuming the appropriate daily nutrients to build overall health to give enough breast milk to their babies. This information should be provided by health personnel during provision of healthcare services such as prenatal care or immunization activities.^31^^,^ 33

Pregnant women faced several health issues, including increased risks of infectious diseases, lack of immediate medical care, and a risk of stress disorder. In particular, pregnant women had difficulty obtaining appropriate medical services, antenatal care, and public health services such as nutrition programs and immunization for children. After the flood in Thailand in 2011, pregnant women did not have access to prenatal care due to difficulties in traveling.^26^ On the other hand, a study on the pregnant women during Hurricane Katrina indicated that most women did not experience difficulty in making prenatal care appointments with other doctors.^17^ After a disaster, there is usually increasing concern about infectious diseases such as cholera, typhoid fever, and measles. Further, disruption of public health services, including immunization for displaced people after disasters, may lead to increased risk of infectious diseases such as measles.^34^^,^ 35 Accordingly, it is important to provide not only acute health services but also to restore regular public health services as swiftly as possible. It is also crucial for national governments to strengthen the capacity of health facilities and to develop disaster health risk reduction and resilience policies for disaster-prone communities.^11^^,^ 36 Intriguingly, pregnant women implemented infection-prevention measures to the best of their knowledge, including boiling all drinking water. Disease prevention is important in both normal circumstances and during disasters, and thus, such preventive practices, including those for their pregnancy, are particularly important for pregnant women. Knowledge on good practices should be provided by health staff and shared among community members.^31^^,^ 33 Such measures might increase community empowerment during extreme events like disasters.

During the acute phase of disasters, while taking all possible actions to survive, mental issues are not considered a priority. Disasters have a substantial impact on the mental health of both pregnant women and their children.^2^^,^ 37 Mothers’ psychiatric problems during pregnancy affect the mental health of their children.^38^^,^ 39 Pregnant women are often in an emotionally insecure state, and they already have depressive tendencies and are prone to mental disorders.^40^ Such mental problems are conducive to adverse effects on child bearing and child development. Thus, it is important to provide adequate mental health support for pregnant women as early as possible. Such an approach may also promote early recovery from trauma for both women and children.

**Limitations**

The study had some limitations. We conducted FGDs in only four communities, and they were conducted about 3–4 months after the typhoon; therefore, the precision of the information provided may have been lacking, particularly with regard to accurate timing and content of assistance, both local and international. As a limitation of the FGDs, we only covered the survivors and could not obtain any information from those who were killed, severely injured, or those who left the community for various reasons. As we considered our purpose, we did not identify the problems and concerns of pregnant women by whether they were multigravidas or primigravidas, nor did we consider differences by trimester and study area. In further studies, it may be necessary to consider these differences in the analysis. The interviewers had no previous experience with FGDs, and only one research group member was familiar with the Waray-Waray language. Moreover, the site selection was not randomly sampled; we used convenient sampling because of a time of social unrest after the disaster.

**Conclusions**

This study investigated women’s concerns during and after the typhoon and presented a first-hand perspective on their problems at the same time. The pregnant women experienced difficulties with evacuation, a lack of minimum survival needs, and attending to their own and their families’ health issues. They used all means possible to survive, including cooperating with neighbors and relatives, using all their existing knowledge, and finding optimal ways to obtain the necessary goods and shelter for survival. It is crucial for pregnant women to receive accurate, rapid, and necessary information before and during the disaster. Community members should be familiarized with the sources of reliable information and methods to obtain such information. In disaster preparation, pregnant women need to understand the signs of premature labor and danger signs of pregnancy such as placenta abruption and pregnancy-induced hypertension, as well as how to handle them under such circumstances. Moreover, health staff should take responsibility for teaching pregnant women the required knowledge about pregnancy issues and relevant coping skills, including protecting children from diseases under disaster circumstances. These points must be addressed in disaster training programs.^41^^,^ 42 The results of this study might be useful to improve preparedness for future disasters and planning for reconstruction and recovery of public health systems after disasters.

## Competing Interests

The authors have no financial relationships or conflicts of interest to disclose.

## Table 1

**Study Area Demographics:** The data were obtained from each RHU

	Municipality X	Municipality Y	Municipality Z
No. of Population	67,966	13,307	20,374
No. of Household	14,757	3,705	4,867
No. of Barangay	33	26	15
Damage of the typhoon			
No. of death	897	4	19
No. of missing	237	0	0
Total household damage	11,607 (78.7%)	2,822 (76.1%)	3,641 (74.8%)
No. of Health Care Facilities			
Governmental Hospital	2	0	0
Rural Health Unit (RHU)	2	1	1
Disaster impact at RHU	Damaged the roofs	Damaged the roofs	Damaged the roofs

## Table 2

**Sample Demographics (N = 53):** 

		M (SD)	N (%)
Age (years)		26.6 (6.72)	
	< 20		8 (15.1)
	20–29		28 (52.8)
	30–39		14 (26.4)
	≥ 40		3 (5.7)
Pregnancy status (person)			
Primigravida			11 (20.8)
Multigravida			42 (79.2)
No. of children		2.6 (2.33)	
0			11 (20.8)
1			9 (17.0)
2			13 (24.5)
3			8 (15.1)
≥4			12 (22.6)
Pregnant status (months)		5.5 (7.58)	
1st trimester			24 (45.3)
2nd trimester			14 (26.4)
3rd trimester			15 (28.3)
House damage (person)			
Total			47 (88.7)
Partial			6 (11.3)

## Appendix 1 Interview Guide for Mothers

1) Could you please explain what happened to you and your family on the day of the typhoon hit, during 1 week, and during 1 month? (The situation of yourself and the family members such as the small children, Immediate needs during or after the typhoon?, Mean of solving)

2) How were the condition of infrastructures, hygiene environment (water and sanitation), nutrition (food supply and clean water), inhabited environment, and economical situation?

3) If you have any health concern and problems of your family members after the typhoon? (Yourself, Your children, Other family members)

4) How about the access to Rural Health Unit or Hospitals? To whom do you contact first? What kinds of support did you receive?

## References

[1] Al Gasseer, N., et al., Status of women and infants in complex humanitarian emergencies. J Midwifery Womens Health, 2004. 49(4 Suppl 1): p. 7-13 10.1016/j.jmwh.2004.05.001 15236698

[2] Dong, X., et al., Depression and its risk factors among pregnant women in 2008 Sichuan earthquake area and non-earthquake struck area in China. J Affect Disord, 2013. 151(2): p. 566-72. 10.1016/j.jad.2013.06.048 23871129

[3] Harville, E.W. and M. Do, Reproductive and Birth Outcomes in Haiti Before and After the 2010 Earthquake. Disaster Med Public Health Prep, 2015: p. 1-8 10.1017/dmp.2015.69 26055727

[4] Liu, S., et al., A report on the reproductive health of women after the massive 2008 Wenchuan earthquake. Int J Gynaecol Obstet, 2010. 108(2): p. 161-4. 10.1016/j.ijgo.2009.08.030 19892335

[5] Antipova, A. and A. Curtis, The post-disaster negative health legacy: pregnancy outcomes in Louisiana after Hurricane Andrew. Disasters, 2015. 10.1111/disa.12125 25754615

[6] Sekizuka, N., et al., Association between the incidence of premature rupture of membranes in pregnant women and seismic intensity of the Noto Peninsula earthquake. Environ Health Prev Med, 2010. 15(5): p. 292-8. 10.1007/s12199-010-0142-5 PMC2921045 21432558

[7] Oni, O., et al., Relationships among stress coping styles and pregnancy complications among women exposed to hurricane katrina. J Obstet Gynecol Neonatal Nurs, 2015. 44(2): p. 256-67. 10.1111/1552-6909.12560 PMC4359646 25712783

[8] Hibino, Y., et al., Health impact of disaster-related stress on pregnant women living in the affected area of the Noto Peninsula earthquake in Japan. Psychiatry Clin Neurosci, 2009. 63(1): p. 107-15. 10.1111/j.1440-1819.2008.01911.x 19154216

[9] Qu, Z., et al., The impact of the catastrophic earthquake in China's Sichuan province on the mental health of pregnant women. J Affect Disord, 2012. 136(1-2): p. 117-23. 10.1016/j.jad.2011.08.021 21937121

[10] EM-DAT The international Disaster Database. [cited 2015 September 20]; Available from: http://www.emdat.be/.

[11] Zakhor, M.J. and D.F. Gillespie, Community disaster vulnerabilities. Theory, Research and Practice. 2013: Springer Science and Business Medical London.

[12] Dolhun, E., Aftermath of Typhoon Haiyan: the imminent epidemic of waterborne illnesses in Leyte, Philippines. Disaster Med Public Health Prep, 2013. 7(6): p. 547-8. 10.1017/dmp.2013.114 24444130

[13] Maplecroft, V. Latest prodcuts and reports. 2014 18 March 2015]; Available from: http://maplecroft.com/profolio/new-analysis/2014/10/29/climate-change-and-lack-food-security-multiply-risks-conflict-and-civil-unreast-32-countries-maplecroft/.

[14] Zhang, S., Haiyan prompts risk research. Nature, 2013. 503: p. 324. 10.1038/503324a 24256787

[15] NDRRMC. NDRRMC Situation Report on the effects of typhoon Yolanda 20th November 2013. 2013 [cited May 20 2014; Available from: http://www.gov.ph/2013/11/20/ndrrmc-situation-report-on-the-effects-of-typhoon-yolanda-november-20-2013-600-a-m/.

[16] World Health Organization, Public health risk assessmnet and interventions Typhoon Haiyan 16 November 2013. 2013: p. 1-31.

[17] Badakhsh, R., E. Harville, and B. Banerjee, The childbearing experience during a natural disaster. J Obstet Gynecol Neonatal Nurs, 2010. 39(4): p. 489-97. 10.1111/j.1552-6909.2010.01160.x PMC2906827 20629936

[18] Callaghan, W.M., et al., Health concerns of women and infants in times of natural disasters: lessons learned from Hurricane Katrina. Matern Child Health J, 2007. 11(4): p. 307-11. 10.1007/s10995-007-0177-4 17253147

[19] Save the children, State of the World's Mothers 2015 Urban disadvantage. 2015: p. 1-80.

[20] Hsieh, H.F. and S.E. Shannon, Three approaches to qualitative content analysis. Qual Health Res, 2005. 15(9): p. 1277-88. 10.1177/1049732305276687 16204405

[21] Zhang, Y. and B.M. Wildemuth, Qualitative Analysis of Content. 2009, CA. USA: Libraries Unlimited Inc.

[22] Warsini, S., et al., Post-traumatic stress disorder among survivors two years after the 2010 Mount Merapi volcano eruption: A survey study. Nurs Health Sci, 2015. 17(2): p. 173-80. 10.1111/nhs.12152 24845603

[23] Kitada, S., et al., The role of a public health nurse drawing local disaster prevention power. Bulletin of the University of Shimane Junior College Izumo Campus, 2011. 5: p. 137-148.

[24] Chan, E.Y.Y., Bottom-up disaster resilience. Nature Geoscience, 2013. 6: p. 327-328.

[25] Yasunari, T., et al., Development and evaluation of 'disaster preparedness' educational programme for pregnant women. Int Nurs Rev, 2011. 58(3): p. 335-40. 10.1111/j.1466-7657.2011.00919.x 21848780

[26] Sanguanklin, N., et al., Effects of the 2011 flood in Thailand on birth outcomes and perceived social support. J Obstet Gynecol Neonatal Nurs, 2014. 43(4): p. 435-44. 10.1111/1552-6909.12466 24956975

[27] Feldman, P.J., et al., Maternal social support predicts birth weight and fetal growth in human pregnancy. Psychosom Med, 2000. 62(5): p. 715-25. 10.1097/00006842-200009000-00016 11020102

[28] The Lancet, The Philippines: learning lessons from past disasters. Lancet, 2013. 382(9906): p. 1679. 10.1016/S0140-6736(13)62406-X 24267984

[29] Annang, L., et al., Photovoice: Assessing the Long-Term Impact of a Disaster on a Community's Quality of Life. Qual Health Res, 2015. 10.1177/1049732315576495 25794525

[30] Persson, M., et al., "Struggling with daily life and enduring pain": a qualitative study of the experiences of pregnant women living with pelvic girdle pain. BMC Pregnancy Childbirth, 2013. 13: p. 111. 10.1186/1471-2393-13-111 PMC3661342 23668823

[31] Ewing, B., S. Buchholtz, and R. Rotanz, Assisting pregnant women to prepare for disaster. MCN Am J Matern Child Nurs, 2008. 33(2): p. 98-103. 10.1097/01.NMC.0000313417.66742.ce 18327108

[32] Davanzo, R., Newborns in adverse conditions: issues, challenges, and interventions. J Midwifery Womens Health, 2004. 49(4 Suppl 1): p. 29-35. 10.1016/j.jmwh.2004.05.002 15236701

[33] Quinn, D., et al., Addressing concerns of pregnant and lactating women after the 2005 hurricanes: the OTIS response. MCN Am J Matern Child Nurs, 2008. 33(4): p. 235-41. 10.1097/01.NMC.0000326078.49740.48 18664905

[34] Kouadio, I.K., et al., Infectious diseases following natural disasters: prevention and control measures. Expert Rev Anti Infect Ther, 2012. 10(1): p. 95-104. 10.1586/eri.11.155 22149618

[35] Murthy, S. and M.D. Christian, Infectious diseases following disasters. Disaster Med Public Health Prep, 2010. 4(3): p. 232-8. 10.1001/dmp.2010.hcn10005 21149220

[36] Giarratano, G., et al., Healthy start: description of a safety net for perinatal support during disaster recovery. Matern Child Health J, 2015. 19(4): p. 819-27. 10.1007/s10995-014-1579-8 PMC4303559 25047787

[37] Landrigan, P.J., et al., Impact of September 11 World Trade Center disaster on children and pregnant women. Mt Sinai J Med, 2008. 75(2): p. 129-34. 10.1002/msj.20032 18500713

[38] Hser, Y.I., et al., Maternal mental health and children's internalizing and externalizing behaviors: Beyond maternal substance use disorders. J Child Fam Stud, 2015. 24(3): p. 638-648. 10.1007/s10826-013-9874-3 PMC4349431 25750503

[39] Madigan, S., et al., Maternal abuse history, postpartum depression, and parenting: links with preschoolers' internalizing problems. Infant Ment Health J, 2015. 36(2): p. 146-55. 10.1002/imhj.21496 25583550

[40] Kirkan, T.S., et al., The depression in women in pregnancy and postpartum period: A follow-up study. Int J Soc Psychiatry, 2015. 61(4): p. 343-9. 10.1177/0020764014543713 25069455

[41] Djalali, A., et al., Art of disaster preparedness in European union: a survey on the health systems. PLoS Curr, 2014. 6. 10.1371/currents.dis.56cf1c5c1b0deae1595a48e294685d2f PMC4323414 25685628

[42] Wolf-Fordham, S., et al., Emergency preparedness of families of children with developmental disabilities: what public health and safety emergency planners need to know. J Emerg Manag, 2015. 13(1): p. 7-18. 10.5055/jem.2015.0213 PMC4487877 25779895

